# *Vibrio vulnificus*: From Oyster Colonist to Human Pathogen

**DOI:** 10.1371/journal.ppat.1006053

**Published:** 2017-01-05

**Authors:** Kelsey E. Phillips, Karla J. F. Satchell

**Affiliations:** Department of Microbiology-Immunology, Feinberg School of Medicine, Northwestern University, Chicago, Illinois, United States of America; Tufts University School of Medicine, UNITED STATES

## Introduction

*Vibrio vulnificus* is a Gram-negative halophilic bacterium commonly found in warm coastal waters. The bacterium can cause severe gastroenteritis from consumption of raw seafood as well as wound infections and necrotizing fasciitis, with mortality rates for sepsis and wound infection at 50% and 17%, respectively [1]. Although infections are rare, *V*. *vulnificus* is responsible for the most deaths caused by *Vibrios* [1] and has the highest per case economic impact of all food-related diseases in the United States [2]. Additionally, the geographical area impacted by *V*. *vulnificus* is expanding due to global warming and rising sea temperatures, such that the incidence of infection has risen worldwide [3]. Even more troublesome, recent studies have identified *V*. *vulnificus* in previously unaffected regions, suggesting there may be future increase of infections [3].

Risk factors for infection include advanced age, male gender, and underlying disease, particularly liver cirrhosis, immunodeficiency, diabetes, and hemochromatosis [1]. However, illness occurs from less than 1 in 10,000 raw oyster meals served to persons with cirrhotic liver, suggesting factors beyond host susceptibility contribute to productive infection [1]. One factor to be considered is whether human-pathogenic *V*. *vulnificus* are relatively rare in food and the environment.

As *V*. *vulnificus* is a diverse bacterial species [4], the specific factor(s) that might define why some isolates cause disease in humans, while the majority of strains are seemingly non-pathogenic, is poorly understood. The identity of these “human-tropic” factors remains the single largest question regarding *V*. *vulnificus* pathogenesis. Discovery and characterization of these factors may then facilitate identifying potentially deadly strains. However, this requires researchers to address critical questions regarding *V*. *vulnificus* human pathogenesis in the context of its natural environment.

### Can Pathogenic Potential be Predicted by Genomes?

For years, researchers have attempted to identify genotypic or phenotypic markers to classify this species into “pathogenic” or “non-pathogenic” strains. However, this has proven difficult due to the nature of the bacterium: *V*. *vulnificus* is naturally competent and frequently exchanges DNA via horizontal gene transfer (HGT), complicating classification systems [4]. One method has been to classify *V*. *vulnificus* into biotypes, with Biotype 1 (BT1) originally composed of human clinical and related environmental strains, Biotype 2 (BT2) containing mostly eel pathogens, and Biotype 3 (BT3) representing a recent outbreak of systemic hybrid strains geographically limited to Israel [5, 6]. BT1 strains were further classified by allelic variation in the gene *vcg* into Clinical (C) and Environmental (E) types [7].

This split of BT1 into two lineages was apparent also by multilocus sequence typing (MLST) [8], with most clinical isolates clustered. However, this linkage of clinical isolates to a single phylogenetic branch has recently been challenged. First, PCR and MLST-based studies showed that *V*. *vulnificus* is in linkage disequilibrium, meaning there is a nonrandom association of alleles at multiple loci [9]. Additionally, BOX-PCR genomic fingerprinting identified 52 unique genotypes, demonstrating that the association between genotype and strain source (environmental or clinical) was not significant. This reveals that genotypic profiles alone are not sufficient predictors of virulence. These authors suggest instead the existence of two different ecotypes, lineages A and B, which represent two major monophyletic groups.

A separate whole-genome SNP analysis of hundreds of clinical and environmental *V*. *vulnificus* strains suggested two ecotypes, A and B [10]. Unexpectedly, BT2 strains previously categorized separately, overlapped with BT1 in the B ecotype, and BT3 strains emerged as an independent branch within the B ecotype. In both the BOX-PCR [9] and whole-genome SNP analyses [10], most of the previously characterized clinical isolates sort to the A lineage or ecotype, but now many sort to the B groupings. Thus, phylogeny does not seem to hold the predictive value for pathogenicity as the number of strains included in these studies expands. Indeed, the genome-wide SNP analysis suggests that it is HGT of chromosomal segments between strains that leads to the continuous evolution of this pathogen [10] and that pathogenesis approaches could be more informative than phylogenetic approaches to understanding *V*. *vulnificus* pathogenicity.

### Classification of *V*. *vulnificus*: A Pathogenesis Perspective?

Because of the abundance of strains and classification systems in addition to an ability to rampantly undergo recombination, development of a rapid identification method, for example in commercial seafood, would be a useful strategy to combat *V*. *vulnificus* infections. There have been several proposed answers to this question.

A widely accepted virulence factor is the large composite MARTX_Vv_ toxin (RtxA1). Due to HGT, the *rtxA1* gene and its encoded toxin can be re-arranged into at least seven different combinations of effector domains, with each effector conferring a distinct cytotoxic activity [11]. Thus, it was considered that a more potent MARTX-type might correlate to human pathogenesis. However, all variants of RtxA1 are capable of lysing host cells, and indeed, a lowered potency toxin was identified in 70% of clinical isolates analyzed [8]. Therefore, it is possible the difference in infectivity between virulent and avirulent strains lies not in their toxin type but in an ability to colonize and survive within the human host.

The capsular polysaccharide (CPS) is a virulence factor that has differential expression between strains. Unencapsulated strains are less virulent than their encapsulated counterparts, which are more invasive in subcutaneous tissue infections than nonencapsulated strains [12]. Furthermore, *V*. *vulnificus* is known to vary expression of CPS via phenotypic conversion, switching from a virulent opaque (Op) colony phenotype to a translucent (Tr) phenotype at a frequency of 10^−3^ to 10^−4^ [13]. However, while CPS has been linked to *V*. *vulnificus* pathogenesis, all strains can switch, except that C-type strains exhibit a significantly lower rate of conversion from Op to Tr phenotypes than E-type strains. This suggests phase variation is more related to environmental survival than to human pathogenicity [14] and is not fully predictive of a virulent phenotype.

*V*. *vulnificus* also variably produces sialic acid-like molecules presented as modifications to the lipopolysaccharide, which likely evolved to facilitate a marine environmental lifestyle. However, these modifications show significant benefit to survival during bloodstream infections and dissemination in vivo [15]. In addition, sialic acid is linked to metabolic fitness in the gut. Recently, *V*. *cholerae* was shown to uptake host-derived sugars, specifically sialic acid, giving this pathogen a competitive advantage in intestinal colonization. This is an important aspect of pathogenicity to be investigated further, especially in pathogens capable of gastrointestinal infection, including *V*. *vulnificus* [16]. On the same theme, sialic acid-like carbohydrates factor into the pathogenicity of *V*. *vulnificus*, since these host-like molecules are required for full motility and biofilm formation, providing a benefit for bacterial survival in the bloodstream [15].

Overall, in comparison to other pathogens, very few factors have been tested for a role in pathogenesis across a broad distribution of strain isolates. Thus, given the genomic diversity and disequilibrium, more research is needed before direct linkage of any single *V*. *vulnificus* virulence factor to human pathogenesis can be postulated as a useful typing scheme to identify virulent versus nonvirulent strains.

### Environmental Pressure for Selection?

**Fig 1:**
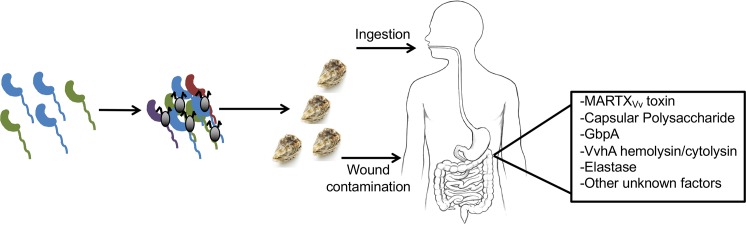
A comprehensive model for *V*. *vulnificus* environmental and pathogenic life cycles. *V*. *vulnificus* exists in coastal waters, where it exchanges genetic material via HGT, continuously forming new strains. Via GbpA, *V*. *vulnificus* binds to chitin in the ocean, which is consumed by filter-feeders such as oysters, which are in turn consumed by humans. Depending on the strain, *V*. *vulnificus* may go on to become pathogenic to humans via several known virulence factors and likely other unidentified factors.

Alternatively, one might consider how this bacterium exists in the environment, as an environmental host is likely the selective force for recurrent emergence of pathogenic strains through HGT. *V*. *vulnificus* produces an *N*-acetylglucosamine (GlcNAc)-binding protein A (GbpA), which mediates bacterial adhesion to chitin [17]. This chitin is consumed by oysters and other filter-feeding shellfish, which in turn accumulates *V*. *vulnificus* and other bacteria [18]. The GbpA protein in *V*. *cholerae* has also been shown to facilitate binding to mucin, which has similar carbohydrate subunits to chitin [19]. Mucin is found lining the human gut and could potentially be a factor for *V*. *vulnificus* colonization. In fact, *V*. *vulnificus* GbpA is also a mucin-binding protein and is essential for pathogenesis in a mouse model [20]. Interestingly, a recent study found E-type *V*. *vulnificus* strains integrate into marine aggregates (chitin) more efficiently than C-type strains. This study also found that expression of *gbpA* was significantly higher in C-type strains [20]. While more research is needed, it is clear that environmental context could be an important link to understanding *V*. *vulnificus* human pathogenesis. Further understanding of selective forces that drive environmental pathogenesis versus commensal colonization of aquatic host could also be useful in discerning the basis of pathogenic emergence (Fig 1).

### Conclusions and Remaining Questions

With the geographical area impacted by *V*. *vulnificus* expanding due to global warming, this bacterium is becoming more important as a human pathogen. While previous classification systems are helpful in classifying strains to biotype, they have not been predictive of pathogenic potential. Overall, an overarching question is how to track human pathogenic strains rapidly enough to detect harmful variants within estuarine waters and seafood? The answer may lie in the continued identification of more virulence factors beyond those already known (Fig 1). This could occur via genome sequencing and subsequent comparative analysis between virulent and avirulent strains and by identification of factors essential for bacterial survival in the environment and environmental hosts. Identification of these genetic differences may lead to rapid identification methods and possible screens. Doing so may drastically impact our ability to detect the human pathogen and subsequently react to its presence.
